# Nitrogen Use Efficiency in Sorghum: Exploring Native Variability for Traits Under Variable N-Regimes

**DOI:** 10.3389/fpls.2021.643192

**Published:** 2021-04-21

**Authors:** Srikanth Bollam, Kirandeep Kaur Romana, Laavanya Rayaprolu, Anilkumar Vemula, Roma Rani Das, Abhishek Rathore, Prasad Gandham, Girish Chander, Santosh P. Deshpande, Rajeev Gupta

**Affiliations:** International Crops Research Institute for the Semi-Arid and Tropics, Patancheru, India

**Keywords:** sorghum, genetic variability, N content, NUE, expression analysis

## Abstract

Exploring the natural genetic variability and its exploitation for improved Nitrogen Use Efficiency (NUE) in sorghum is one of the primary goals in the modern crop improvement programs. The integrated strategies include high-throughput phenotyping, next generation sequencing (NGS)-based genotyping technologies, and *a priori* selected candidate gene studies that help understand the detailed physiological and molecular mechanisms underpinning this complex trait. A set of sixty diverse sorghum genotypes was evaluated for different vegetative, reproductive, and yield traits related to NUE in the field (under three N regimes) for two seasons. Significant variations for different yield and related traits under 0 and 50% N confirmed the availability of native genetic variability in sorghum under low N regimes. Sorghum genotypes with distinct genetic background had interestingly similar NUE associated traits. The Genotyping-By-Sequencing based SNPs (>89 K) were used to study the population structure, and phylogenetic groupings identified three distinct groups. The information of grain N and stalk N content of the individuals covered on the phylogenetic groups indicated randomness in the distribution for adaptation under variable N regimes. This study identified promising sorghum genotypes with consistent performance under varying environments, with buffer capacity for yield under low N conditions. We also report better performing genotypes for varied production use—grain, stover, and dual-purpose sorghum having differential adaptation response to NUE traits. Expression profiling of NUE associated genes in shoot and root tissues of contrasting lines (PVK801 and HDW703) grown in varying N conditions revealed interesting outcomes. Root tissues of contrasting lines exhibited differential expression profiles for transporter genes [ammonium transporter (*SbAMT)*, nitrate transporters (*SbNRT*)]; primary assimilatory (glutamine synthetase (*SbGS)*, glutamate synthase (*SbGOGAT[NADH], SbGOGAT[Fd])*, assimilatory genes [nitrite reductase (*SbNiR[NADH]3*)]; and amino acid biosynthesis associated gene [glutamate dehydrogenase (*SbGDH*)]. Identification and expression profiling of contrasting sorghum genotypes in varying N dosages will provide new information to understand the response of NUE genes toward adaptation to the differential N regimes in sorghum. High NUE genotypes identified from this study could be potential candidates for in-depth molecular analysis and contribute toward the development of N efficient sorghum cultivars.

## Introduction

Sorghum [*Sorghum bicolor* (L.) Moench] is one of the important staple food crops and fifth-most cultivated cereal after wheat, rice, maize, and barley (Taylor et al., 2006). Sorghum is a multi-purpose commodity in terms of its utility, such as grain sorghum for human food and animal feed, forage sorghum for forage and fodder, sweet stalk sorghum providing fiber, and feedstock for biofuel. Genetic and phenotypic variability in sorghum is evident by its spread from the North America to the African continent, through the Middle East, the Indian sub-continent, and further parts of East Asia to Australia resulted in distinct botanical races (Kimber, 2000; Morris et al., 2013). Sorghum followsC_4_ type photosynthesis pathway, and its efficient use of nutrients, radiation, and water makes it adaptable to harsh and water-limited conditions (Paterson et al., 2008). Due to cultivated sorghum’s small genome size (812 Mbp) and diploid nature (2*n* = 20), sorghum is used as a model for genome analysis. The deep root system architecture of sorghum makes it drought-tolerant and adaptable to grow in a water-limited environment. Despite its C_4_ nature and relatively better drought tolerance compared to maize (Paterson et al., 2009), sorghum still depends mainly on nitrogen (N) fertilizer for achieving higher grain yields in an intensive agricultural system. Nitrogen is an essential macronutrient, most abundantly absorbed by roots, and 75% of the N present in the leaf is allocated to chloroplasts. N is the primary constituent of most of the important biomolecules *viz*., nucleotides, amino acids, proteins, and hormones related to the plants overall growth and development. About 1.5–2.0% of total plant dry matter and 16% of the plant protein was covered by N (Frink et al., 1999).

In the last four decades, breeding efforts, along with the use of synthetic N fertilizers, enabled substantial increment in crop productivity, especially in irrigated production systems, contributing to the “Green Revolution” that addressed the global food needs. However, 50–70% of applied nitrogen fertilizer lost to the environment through volatilization, leaching, groundwater runoff, and nitrous oxide emissions from N fertilizer residues, in-turn, pose adverse effects on the environment (Zhang et al., 2013). High fertilizer N application is one of the major input costs to farmers, and it also affects soil health by acidification. With the priority of lower N fertilizer input and environmentally friendly agriculture (in the intensive production systems), the development of crops and/or genotypes with high NUE (especially in the subsistence farming) and better yield is critical for the sustainable production of sorghum across diverse agroecosystems in the world. NUE in crop plants is a very complex phenomenon, governed by the economic produce of the species (grain, forage/fodder, or dual-purpose) and is defined as the quantity of biomass and/or grain produced per unit of available N in the soil (Moll et al., 1982; Good et al., 2004). Crop response to N mainly depends on the genotype and its interaction with applied N fertilizer (Masclaux-Daubresse and Chardon, 2011). Under the high N conditions, most commonly observed in the intensive agricultural systems, variation in NUE is primarily attributed to differences in N uptake capacity. In contrast, under limited N conditions, prevalent in sorghum production areas in Asia and Africa, NUE variation is driven by changes in N remobilization and utilization efficiency. There is a huge genetic variability is present for NUE, and associated traits in cereals [sorghum (Youngquist et al., 1992), rice (Tirol-Padre et al., 1996; Rao et al., 2018), wheat (Le Gouis et al., 2000), and maize (McCullough et al., 1994)]. This genetic variability is a valuable source to help understand physiological, molecular, and genetic basis of NUE and its further exploitation for the development of high NUE crops.

NUE is a complex trait and is driven by many genes associated with N uptake, assimilation, and remobilization. A comprehensive understanding of physiological and molecular mechanisms under pinning N stress tolerance and/or NUE in sorghum is critical for its effective exploitation, facilitated through available genetic and genomic resources. By utilizing the next generation sequencing (NGS) technologies and gene identification strategies, putative genes associated with NUE in sorghum were identified and characterized (Gelli et al., 2014, 2016, 2017; Massel et al., 2016; Diatloff et al., 2017). However, very few studies with expression studies at varying N conditions in different tissue samples are conducted. In the present study, we have conducted a series of field and lab experiments to evaluate the genotype, nitrogen treatment (0, 50, and 100% of the recommended N) and season (two different seasons) specific variations associated with NUE in sixty diverse sorghum accessions. Wide variability was observed for physiological, agronomical, growth, and biological yield parameters associated with NUE in sorghum. We have generated genotyping by sequencing (GBS) based single nucleotide polymorphism (SNP) data to study the genetic diversity for this panel of sorghum genotypes. High and low NUE genotypes were identified based on their performance under low N conditions. We have also identified grain, fodder and dual-purpose sorghum lines which could be potential donors for crop improvement programs. We also studied the expression profiles of NUE associated genes in shoot and root samples of contrasting sorghum genotypes under varying N conditions.

## Materials and Methods

### Plant Material

The plant material included a diverse set of 60 sorghum accessions (Supplementary Table 1). This set included parents of mapping populations such as Back-Cross derived Nested Association Mapping (BCNAM) populations; bi-parental mapping populations, and accessions from different countries such as India, Niger, Sudan, South Africa, Pakistan, Yemen, Cameroon, Nigeria, United States, Lesotho, Ethiopia, Mali, and the United States.

### Experimental Design

The set of 60 sorghum accessions were field evaluated in a split-plot alpha lattice design under three N fertilizer application levels (0, 50, and 100% of the recommended (90 kg ha^–1^) N) with three replications for two seasons (2016–17 and 2017–18) in the black soil precision fields of International Crops Research Institute for the Semi-Arid Tropics (ICRISAT), Patancheru, India. Before starting the experiment, soil testing was done by collecting samples from 0 to 12 inches, and 12–24 inches, across the field as per the standard sampling procedure. The results were provided in Supplementary Table 2. An individual evaluation test plot with 2 m length and four-rows with 0.60 m inter-row spacing were sown at a density of 15–20 seeds (with 0.15 m plant to plant distance) per row for each accession. All entries were tractor planted on the same day in tilled plots. Other fertilizers such as phosphorus (P) and potassium (K) were applied to all plots at the rate of 50 and 40 kg ha^–1^, respectively (as per the recommendation of PJ Telangana State Agricultural University, Rajendra Nagar, Hyderabad for the trial production ecology). The basal application of P and K fertilizers except N was applied in 2 equal splits at 20 days from the date of emergence and 1 month after application of the first N dose. The sources of N, P, and K were urea (46% N), single superphosphate (16% P_2_O_5_), and murate of potash (60% K_2_O). Weeding was done at 20–30 days interval. During 2016–17, sowing was done on 15th December 2016 and the trial was harvested on 27th April 2017. Similarly, during 2017–18, sowing took place on 17th November 2017 and the trial was harvested on 4th April 2018. Irrigation was given four times in each season during 2016–17 (16th December 2016, 29th December 2016, 18th January 2017, and 02nd February 2017) and 2017–18 (18th November 2017, 04th December 2017, 20th December 2017, 05th January 2018) (Supplementary Figures 1, 2).

### Phenotyping of the Traits Associated With NUE

Different physiological, agronomical, and biological yield attributes associated with NUE were systematically recorded in three different N dosages (0, 50, and 100% of the recommended N) for two seasons^1^.

#### Leaf Parameters

Chlorophyll content (CC) was recorded on the flag leaf of three random plants of the middle two rows of the plot using SPAD meter (Konica Minolta Sensing Americas, Inc., Ramsey, NJ) at the anthesis stage (around 75 days after emergence). Leaf area (LA); specific leaf area for each genotype was measured by running all the harvested leaves of a plant through benchtop leaf area meter Li3100C (LI-COR Inc., Lincoln, NE, United States). Leaf number (LN); where leaf number of each genotype harvested for LA were counted.

#### Growth Parameters

During flowering stage, days to 50% flowering (DFL); number of days from the emergence date to the day on which 50% of the plants in a plot reached anthesis at least halfway. At the harvest stage, Plant height (PH) was measured from the base of the plant to the tip of the main head as an average of three plants, randomly chosen from the center two rows of each plot and expressed in cm. Plant stand (PS); the total number of plants in the center two rows of each plot was recorded. Number of tillers (NT); the total number of tillers in the center two rows of each plot were recorded.

#### Panicle Parameters

Panicle number (PN); the total number of heads in the center two rows of each plot were recorded. Panicle weight (PW); weight (g/plot) of all the panicles in the center two rows of each plot were measured.

#### Biological Yield Parameters

Fresh straw yield (FSY); weight (g/plot) of freshly harvested straw was recorded for center two rows of each plot. Dry straw yield (DSY); weight (g/plot) of 10 days sundried straw was recorded for center two rows of each plot. Grain yield (GY); grain weight (g/plot) of all the panicles which are sun-dried and threshed from the center two rows of each plot. Test weight (TW); 200 seed weight (g) of each genotype was recorded. Harvest Index [HI (%)] is the ratio of grain yield to the total biomass, considered as the measure of biological success.

#### NUE Parameters

N content in grain (GN%) and N content in straw (SN%) of each genotype was estimated by sulfuric acid-selenium digestion method (Sahrawat et al., 2002). Grain and starw samples were fine powdered using clone mixture (Cyclone sample mill), 250 mg of fine powder was used for N estimation at Charles Renard Analytical laboratory, ICRISAT. The samples were digested with sulfuric acid-selenium and then analyzed using an Auto-analyzer (Skalar SAN System, AA Breda, Netherlands).

### Genotyping-by-Sequencing and Single Nucleotide Polymorphism Identification

DNA was isolated from leaves of each accession at 4–6 leaf stage using the modified hexadecyltrimethyl ammonium bromide (CTAB) protocol (Mace et al., 2003). Genotyping was performed by following the GBS approach (Elshire et al., 2011), restriction enzyme *Ape*KI (NEB R0643L) used for complexity reduction. The GBS library was sequenced on IlluminaHiSeq 2500 (Illumina Inc., San Diego, CA, United States) following the manufacturer’s protocol. SNPs were called using the TASSEL v5.2 GBS pipeline (Bradbury et al., 2007) against sorghum assembly v3.1 (Phytozome.JGI.doe.gov. Phytozome, 2019). The final data included a total of 89,770 SNPs (obtained from 58 accessions out of 60) with minor allele frequency (MAF) > 1%, and missing data <50% were used in this study.

### Diversity and Population Structure

An unweighted neighbor-joining phylogenetic tree was constructed in TASSEL v5.2 (TASSEL v5.2). The hierarchical population structure was estimated by using the ADMIXTURE program, a model-based estimation of ancestry in unrelated individuals using the maximum-likelihood method (Alexander et al., 2009). ADMIXTURE implements a cross-validation (CV) feature, together with the number of iterations to convergence, allowing to determine the number of subpopulations (*k*-values) that best fits the data. The Admixture analysis was performed for different K (number of sub-populations) varying from 2 to 8. The most appropriate *K*-value was selected after considering 10-fold cross-validations whereby the best K exhibits low cross-validation error compared to other *K*-values and good correspondence with the clustering pattern obtained by hierarchical cluster tree.

### Contrasting Genotypes Screening for N Stress Under Hydroponics System and Tissue Sample Collection for Expression Studies

**FIGURE 1:**
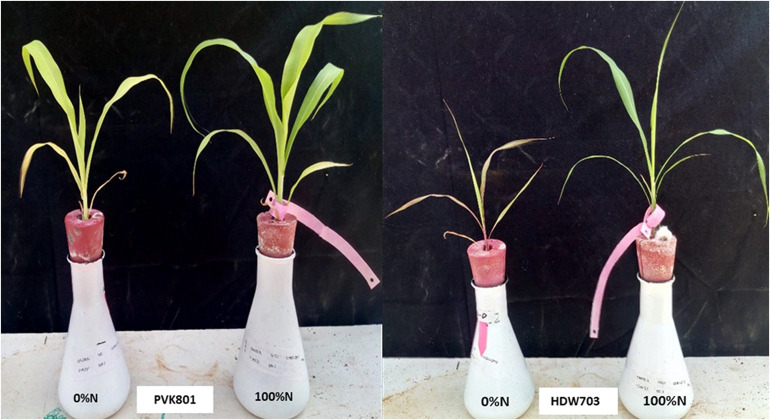
High NUE/better (PVK801) and low NUE/poor (HDW703) performers grown under varying N (N0 and N100) conditions in hydroponics system using modified Hoagland solution.

Based on grain yield data under low N conditions, two contrasting genotypes for NUE (High NUE/better performer: PVK801 and low NUE/poor performer: HDW703) were selected for expression profiling. Sorghum accessions were germinated on the sand. Eight-days-old seedlings with uniform length (both plumule and radicle) were selected and transferred to the nutrient solution (Modified Hoagland) in the glasshouse. The seedlings were maintained under a 16/8 h photo-period cycle at 25°C (day) and 18°C (night). The pH of the nutrient solution adjusted to 5.8 and refreshed every 3 days. Two weeks old seedlings were transferred to a modified Hoagland solution with 0 and 100% of recommended N conditions. The plants grown in 0% N generated N stress symptoms. From 24 days old seedlings (Figure 1), shoot and root samples were collected separately and frozen in liquid nitrogen and stored at −80°C until RNA isolation.

### Total RNA Isolation, cDNA Synthesis, and Primer Design

Total RNA was isolated from the shoot and root tissues using the RN easy Plant Mini kit (Qiagen, Germany). The quality of the isolated RNA was determined by Nanodrop^®^ ND100 spectrophotometer (Thermo Fisher Scientific, United States) and RNA was treated with RNAse free DNAse enzyme (Thermo Fisher Scientific, United States). One microgram of total RNA was used for first-strand cDNA synthesis using the Superscript III RT kit (Life Technologies, United States) as per the manufacturer’s instructions. A total of 13 genes associated with N uptake, assimilation, and remobilization were selected *a priori*, based on previously published studies. Gene-specific primers were designed (Supplementary Table 3) using Primer3 software^2^ with the specifications such as—G + C content 45–55%, Amplicon size 80–170 base pairs (bp), length of primer 19–24 nucleotides and melting temperature (Tm) 58–62°C.

### Quantitative Real-Time PCR (qRT-PCR) Analysis

All the qRT-PCR reactions were performed on Applied Biosystems 7500 Real-Time PCR (Life Technologies, United States), in 96 well optical plates. Reactions were performed in a final volume of 20 μl, containing 60 ng of cDNA samples, 10 μl of SYBR^®^ Green qPCR SuperMix (Invitrogen, United States), 500 nM of each primer. The qRT-PCR cycling conditions included: 50°C for 2 min (Pre-incubation), 95°C for 10 min (denaturation) followed by 40 cycles of 95°C for 15 s (denaturation), 60°C for 1 min (annealing and extension). For all the samples, qRT-PCR was performed on three biological replicates and three technical replicates. Controls were set up for each sample in duplicates using the *SbUbiquitin* gene (internal control) for normalizing the gene expressions. qRT-PCR data were analyzed by 7500 Sequence Detection Software (Applied Biosystems, United States) with default baseline and threshold, relative expression of genes calculated by the 2^–Δ*CT*Δ*CT*^ method (Schmittgen and Livak, 2008).

### Statistical Analysis

Analysis of variance (ANOVA) for all the traits was performed for individual and across seasons using PROC MIXED procedure of SAS version 9.4 (SAS Institute Inc [SAS], 2018), considering the season, treatments (whole plot), genotype (subplot), replications, as fixed effects and block as a random effect. Individual season variances were modeled into the combined analysis. Best Linear Unbiased Estimates (BLUE’s) were calculated for the main and interaction effects of season, treatment, and genotype. Multiple comparisons were performed for significant effects (*p* < 0.05). Correlation coefficient analysis was performed using the PROC CORR procedure.

## Results

### Evaluation of Sorghum Genotypes in Field Conditions for Two Seasons

#### Effects of Nitrogen and Genotypes on Leaf Parameters

The post-flowering SPAD showed an increase in mean and range values among N application rates from 0 to 100 in both individual seasons and pooled data with 11–16% variation (Table 1). The ANOVA revealed highly significant genotype × treatment interaction for the season 2017 and pooled data. A gradual increase in leaf area was observed in 2017 and across seasons, but a slight decrease was observed for the N50 treatment in 2016. The genotype × treatment was significant in individual and pooled seasons along with significant interaction effects for season × genotype and season × genotype × treatment. An exponential increase in leaf number with N dosage in individual seasons and across seasons with significant variance for season × genotype (Table 2) was observed.

**TABLE 1:** Summary performance of 60 sorghum genotypes grown in different N conditions (0 N, 50% N, and 100% of the recommended N) for the season 2016–17, 2017–18, and across seasons.

		**2016–17**		**2017–18**		**Across seasons**	
**Trait**	**Treatment**	**Range**	**Mean**	**Range**	**Mean**	**Range**	**Mean**
**SPAD (at anthesis stage)**	N0	29.23–47.10	38.54	16.73–39.86	28.33	26.12–41.30	33.44
	N50	34.55–50.05	43.13	23.58–47.71	33.32	31.91–46.55	38.23
	N100	41.54–55.15	48.89	27.47–56.42	40.85	36.84–53.87	44.88
**Leaf Area (cm^2^)**	N0	643.50–6459.72	2055.33	599.43–3187.43	1644.83	706.13–4486.14	1850.08
	N50	587.28–3408.67	1881.42	669.93–4212.95	2082.35	944.93–3431.58	1981.89
	N100	1126.86–8967.24	2444.70	928.84–5479.43	2345.36	1221.04–6082.23	2395.03
**Leaf count (no.)**	N0	6.35–26.93	12.78	7.13–21.90	12.10	8.23–23.13	12.44
	N50	6.48–21.22	11.52	7.00–26.00	13.58	7.49–21.68	12.53
	N100	7.82–26.21	12.72	8.26–39.83	16.60	8.88–25.11	14.60
**Number of panicles**	N0	16.17–131.27	29.059	16.30–198.43	37.31	19.02–107.00	33.07
	N50	17.10–52.97	31.51	17.36–161.73	42.21	19.26–99.95	36.67
	N100	21.98–126.78	35.34	19.63–91.81	43.35	21.94–108.59	39.24
**Wt. of panicles (g)**	N0	216.97–1847.38	887.77	174.38–1347.77	590.93	382.68–1325.59	739.35
	N50	282.29–1820.03	998	265.39–1827.33	847.45	372.91–1657.17	922.89
	N100	617.00–2323.72	1393.78	371.06–1951.89	924.23	600.47–1734.42	1159.00
**Grain yield (g)**	N0	125.54–1460.42	641.09	13.42–1159.42	359.40	195.14–972.92	500.24
	N50	179.35–1382.67	742.36	48.11–1244.52	555.09	169.73–1218.68	649.17
	N100	245.58–1879.09	1061.09	25.23–1355.34	556.47	147.42–1569.09	809.19
**Test weight (g)**	N0	0.78–3.98	2.56	0.90–4.15	2.54	0.84–4.03	2.55
	N50	1.03–3.98	2.64	1.29–4.43	2.72	1.16–4.14	2.68
	N100	1.02–4.01	2.74	1.20–4.64	2.73	1.18–4.31	2.74
**Fresh stalk yield (kg)**	N0	2946.28–9832.02	5442.16	1873.04–6797.77	4349.18	2842.85–7850.58	4895.67
	N50	3876.03–11764.00	6387.30	2626.82–10838.00	5677.98	3379.89–11205	6032.62
	N100	4223.51–16276.00	7808.41	2175.64–10654.00	5819.94	3199.58–12943.00	6814. 17
**Dry stalk yield (kg)**	N0	1481.06–4522.73	2980.12	1071.46–3938.98	2273.48	1583.76–4038.23	2626.80
	N50	2169.29– 6394.11	3348.63	1267.65–6009.78	2858.36	1755.07–5598.91	3103.50
	N100	2284.02– 6476.93	3878.90	1207.92–5080.20	2956.85	1916.44–5712.39	3417.88
**Harvest index**	N0	3.87–42.75	19.99	1.05–50.45	15.38	5.84–35.86	17.68
	N50	6.73–39.48	21.48	0.10–60.99	19.70	5.40–40.85	20.95
	N100	7.60–50.89	26.61	0.26–63.21	20.42	6.85–49.16	23.16
**N content in grain**	N0	1.17–2.12	1.63	1.05–2.25	1.37	1.20–2.09	1.50
	N50	1.16–2.24	1.65	0.90–1.93	1.35	1.04–1.89	1.50
	N100	1.18–2.18	1.64	1.13–1.90	1.46	1.23–1.90	1.55
**N content in stalk**	N0	0.40–1.36	0.72	0.19–0.64	0.38	0.32–0.96	0.55
	N50	0.38–1.25	0.76	0.17–0.72	0.34	0.31–0.90	0.55
	N100	0.38–1.18	0.73	0.21–0.94	0.45	0.32–0.90	0.59

**Data presented here is the mean of the three replications.**

**TABLE 2:** Summary of ANOVA for leaf, growth, panicle, biological yield parameters along with NUE traits under three N dosages in field for the season 2016–17, 2017–18, and across seasons.

**S. No.**	**Trait**	**2016–17**		**2017–18**		**Across seasons**						
		**Genotype (G)**	G * T	**Genotype (G)**	G * T	**Genotype (G)**	**Treatment (T)**	**Season (S)**	G * T	S * G	S * T	S * G * T
**Leaf parameters**												
1	SPAD (at anthesis stage)	2.16	0.81^n s^	10.99	5.81	7.03	51.81	14.26^n s^	2.48	4.1	0.53	2.96
2	Leaf Area (cm^2^)	10.71	2.92	11.27	3.75	15.74	37.23	0.16^n s^	3.19	5.91	11.14	3.54
3	Leaf count (no.)	3.13	0.97^n s^	5.02	1.68^n s^	5.19	2.60^n s^	1.13	1.27^n s^	2.26	2.22	1.25^n s^
**Growth parameters**												
1	Days to 50% flowering (days)	23.22	0.92^n s^	37.27	1.42	36.34	0.31^n s^	0.45^n s^	1.15^n s^	6.49	1.83	1.03^n s^
2	Plant height (cm)	76.86	1.22^n s^	176.9	13.8	192.18	2.35^n s^	1329.51	6.61	12.38	0.89	6.37
3	Number of tillers	7.87	1.02^n s^	17.95	1.86	21.6	14.98^n s^	3847.82	1.63^n s^	5.01	1.28	1.53^n s^
**Panicle parameters**												
1	Number of panicles	9.2	1.54^n s^	0.97^n s^	1.01^n s^	8.79	20.95^n s^	163.30^n s^	1.04^n s^	2.04	1.21	1.26
2	Wt. of panicles (g)	4.52	1.53^n s^	26.75	4.08	13.42	249.81	14.02	3.58	8.6	36.51	3.43
**Biological yield parameters**												
1	Grain yield (g)	4.81	1.52^n s^	40.31	5.66	23.01	118.46	23.09^n s^	3.41	11.25	34.05	4.28
2	Test weight (g)	6.23	3.82	31.36	5.47	82.23	38.7	0.24^n s^	4.33	11.78	3.71	3.62
3	Fresh stalk yield (kg)	10.02	1.47^n s^	27.24	3.28	24.1	39.77	8.58^n s^	2.48	6.04	4.61	1.85
4	Dry stover yield (kg)	11.41	1.08^n s^	22.66	2.49	25.67	29.43^n s^	536.84^n s^	1.92	4.5	2.16	1.62^n s^
5	Harvest Index	8	1.09^n s^	29.81	2.43	24.64	12.99^n s^	2.14	1.66^n s^	12.39	3.75	2
**NUE traits**												
1	N content in grain	8.63	1.12^n s^	11.29	1.76	9.48	1.01^n s^	3.89^n s^	1.52^n s^	9.73	0.99	1.19^n s^
2	N content in stalk	2.75	1.5^n s^	13.04	5.32	5.58	6.91^n s^	30515^n s^	2.99	3.64	14.45	2.5

**“ns” denotes for not significant. All Fstat/genetic variance are significant @p of 0.001 except for traits with “ns.”**

#### Effects of Nitrogen and Genotypes on Growth Parameters

The flowering time exhibited a minimum variation with N treatments in both individual and pooled seasons, with genotype x treatment interaction being non-significant (*p* ≥ 0.05) except during the 2017 trial. A gradual increase in plant height was observed with increasing dosage of N with significant genotype × treatment, season × genotype, and season × genotype × treatment interactions in pooled seasons. The number of tillers increased with a higher quantity of N in both individual and pooled seasons, and significant genotype × treatment interaction was noted for 2017 (Table 2).

#### Effects of Nitrogen and Genotypes on Panicle Parameters

With increase in dosage of nitrogen, panicle number also increased in individual and across seasons with significant season × genotype and season × genotype × treatment interactions. Panicle number observed more than 15% increase at N50 compared to N0 in individual seasons (Table 1). Accordingly, in both individual and across seasons, the panicle weight also increased with higher N concentrations. Significant genotype × treatment, season × genotype and season × genotype × treatment interactions were noted (Table 2).

#### Effects of Nitrogen and Genotypes on Biological Yield Parameters

Both grain yield and test weight exhibited an increasing trend with higher quantities of N dosages with 15–20% variation for grain yield. Significant interactions for genotype × treatment, season × genotype and season × genotype × treatment interactions were observed except in 2016 for grain yield.

In individual seasons fresh biomass yield recorded gradual increase with higher N-dosages, but the magnitude of increase from N0 to N50 was more compared to N50 to N100 dosages. The genotype × treatment, season × genotype and season × genotype × treatment interactions were significant overall except for 2016. The same trend was observed for dry stover yield where a gradual increase in weight was observed in individual seasons. A wide range of 11–24% variation was observed between N0 to N50 treatments in all seasons (Table 1). Harvest index had a steady increase with increasing dosages of nitrogen with season × genotype and season × genotype × treatment significant (Table 2).

#### Effects of Nitrogen and Genotypes on NUE Parameters

The grain N content plunged from N0 to N50, while it increased from N50 to N100 in individual and across seasons (Table 1). The stalk N content was similar in range for N0 and N50 across seasons, whereas in 2017, there was a decrease from N0 to N50 and a rise to N100 treatments. All the interaction effects were significant, except in2016 for stalk N. Genotype × treatment interactions are significant in 2017 for grain N content (Table 2).

### Correlation Coefficient Analysis

The correlation coefficient analysis with three different N treatments revealed a significant positive correlation between leaf area and flowering time in individual and across seasons. Under N0, N50, and N100 treatments, fresh and dry biomass yield had significant correlations with flowering time and leaf area except for 2017 under N100 treatment (*p* ≥ 0.05). Plant height was also positively correlated with biomass yields except in the 2017 trial under N0 and N100 conditions. In the 2017season, biomass yields were negatively correlated with grain yield and panicle weight except in N50 dosages (*p* ≥ 0.05). Under all treatments, fresh biomass had a significant positive correlation with dry biomass yield in individual and across seasons. A similar trend was observed for panicle weight with grain yield; and the number of tillers with panicle number and leaf number. Grain N percent had significant negative correlations to panicle weight and grain yield (except for N0 treatment for gain yield). Stalk N content was positively correlated with grain N content and had a negative correlation with plant height (except for N100 with prob ≥ 0.05). A significant positive correlation was observed between stalk N content and panicle number under N0 treatment in 2017 and across seasons (Tables 3–5 and Supplementary Tables 4, 5).

**TABLE 3:** Correlation coefficient analysis of 60 genotypes for different traits at N0 condition across seasons.

**Trait**	**DFL**	**CC**	**LA**	**PHT**	**PN**	**GY**	**PW**	**DSY**	**FSY**	**LN**	**NT**	**HI**	**TW**	**GN%**	**SN%**
**DFL**	1														
**CC**	0.16	1													
**LA**	0.72**	0.28*	1												
**PHT**	0.01	−0.23	−0.05	1											
**PN**	−0.28*	−0.11	−0.34**	−0.05	1										
**GY**	−0.29*	−0.05	−0.29*	−0.08	−0.02	1									
**PW**	−0.09	0.05	−0.28*	0.04	0.04	0.68**	1								
**DSY**	0.56**	0.06	0.43**	0.48**	−0.27*	−0.28*	−0.13	1							
**FSY**	0.59**	0.12	0.38**	0.42**	−0.21	−0.3*	−0.14	0.92**	1						
**LN**	0.27*	0.07	0.36**	0.07	0.34**	−0.35**	−0.34**	0.16	0.19	1					
**NT**	−0.1	0.03	−0.11	−0.04	0.52**	−0.16	−0.06	−0.15	−0.05	0.52**	1				
**HI**	−0.38**	−0.16	−0.41**	−0.17	−0.02	0.87**	0.62**	−0.58**	−0.55**	−0.43**	−0.09	1			
**TW**	−0.12	0.14	0.05	−0.08	−0.38**	0.2	0.19	−0.06	−0.15	−0.2	−0.29*	0.22	1		
**GN%**	0.21	0.18	0.28*	−0.04	−0.18	–0.34**	−0.14	0.17	0.1	0.06	0.02	−0.27*	0.03	1	
**SN%**	0.08	−0.07	0.13	−0.28*	0.39**	−0.05	−0.18	−0.21	−0.2	0.18	0.24	−0.02	−0.07	0.26*	1

*DFL, Days to 50% flowering; CC, SPAD at anthesis stage; LA, Leaf area; PHT, Plant height; PN, Panicle number; GY, Grain yield (g); PW, Panicle weight (g); DSY, Dry stalk yield (g); FSY, Fresh stalk yield (g); LN, Leaf number; NT, Number of tillers; HI, Harvest index; TW, Test weight (100 seed weight); GN%, N content in grain, SN%, N content in stalk.* * denotes p = 0.05, ** denotes p = 0.001.

**TABLE 4:** Correlation coefficient analysis of 60 genotypes for different trait at N50 condition across seasons.

**Trait**	**DFL**	**CC**	**LA**	**PHT**	**PN**	**GY**	**PW**	**DSY**	**FSY**	**LN**	**NT**	**HI**	**TW**	**GN%**	**SN%**
**DFL**	1														
**CC**	−0.05	1													
**LA**	0.65**	0.02	1												
**PHT**	0.02	−0.14	0.08	1											
**PN**	−0.29*	−0.18	−0.36**	−0.01	1										
**GY**	−0.09	0.02	−0.02	−0.23	−0.09	1									
**PW**	0.003	−0.06	0.05	−0.23	−0.16	0.84**	1								
**DSY**	0.43**	−0.05	0.47**	0.5**	−0.13	−0.25	−0.15	1							
**FSY**	0.41**	−0.0004	0.38**	0.44**	−0.03	−0.3*	−0.2	0.93**	1						
**LN**	0.16	−0.09	0.29*	−0.001	0.39**	−0.12	−0.2	0.17	0.17	1					
**NT**	−0.11	−0.13	−0.11	−0.01	0.67**	−0.06	−0.1	0.09	0.17	0.58**	1				
**HI**	−0.24	−0.06	−0.25	−0.31*	−0.08	0.85**	0.71**	−0.6**	−0.62**	−0.35**	−0.18	1			
**TW**	−0.19	0.15	0.1	0.04	−0.47**	0.16	0.29*	0.01	−0.11	−0.16	−0.3*	0.11	1		
**GN%**	0.11	0.06	0.003	0.22	−0.07	−0.38**	−0.31*	0.11	0.1	−0.09	−0.09	−0.39**	−0.12	1	
**SN%**	0.03	0.15	0.06	−0.31*	−0.11	−0.08	−0.05	−0.09	−0.06	−0.05	−0.14	−0.07	−0.04	0.34**	1

*DFL, Days to 50% flowering; CC, SPAD at anthesis stage; LA, Leaf area; PHT, Plant height; PN, Panicle number; GY, Grain yield (g); PW, Panicle weight (g); DSY, Dry stalk yield (g); FSY, Fresh stalk yield (g); LN, Leaf number; NT, Number of tillers; HI, Harvest index; TW, Test weight (100 seed weight); GN%, N content in grain, SN%, N content in stalk.* * denotes p = 0.05, ** denotes p = 0.001.

**TABLE 5:** Correlation coefficient analysis of 60 genotypes for different traits at N100 condition across seasons.

**Trait**	**DFL**	**CC**	**LA**	**PHT**	**PN**	**GY**	**PW**	**DSY**	**FSY**	**LN**	**NT**	**HI**	**TW**	**GN%**	**SN%**
**DFL**	1														
**CC**	−0.15	1													
**LA**	0.54**	−0.14	1												
**PHT**	0.17	−0.21	0.08	1											
**PN**	−0.28*	−0.12	−0.29*	0.15	1										
**GY**	−0.05	−0.16	−0.24	−0.02	0.1	1									
**PW**	0.02	−0.1	−0.26*	−0.19	−0.01	0.84**	1								
**DSY**	0.51**	−0.29*	0.48**	0.38**	−0.06	−0.19	−0.17	1							
**FSY**	0.5**	−0.22	0.45**	0.36**	−0.01	−0.06	0.002	0 9**	1						
**LN**	0.06	−0.22	0.39**	0.13	0.37**	−0.31*	−0.26*	0.16	0.21	1					
**NT**	−0.34**	−0.17	−0.13	−0.02	0.73**	−0.15	−0.15	−0.08	−0.11	0.49**	1				
**HI**	−0.3*	−0.01	−0.35**	−0.12	0.02	0.79**	0.66**	−0.64**	−0.5**	−0.35**	−0.15	1			
**TW**	−0.11	0.05	−0.09	−0.08	−0.42**	0.09	0.15	−0.01	0.003	−0.25	−0.39**	0.02	1		
**GN%**	0.07	0.22	0.28*	−0.01	−0.1	−0.4**	−0.4**	0.004	−0.005	0.05	0.01	−0.32*	−0.11	1	
**SN%**	0.07	0.23	−0.01	−0.11	−0.08	−0.21	−0.12	−0.05	0.01	−0.13	−0.18	−0.09	−0.04	0.19	1

*DFL, Days to 50% flowering; CC, SPAD at anthesis stage; LA, Leaf area; PHT, Plant height; PN, Panicle number; GY, Grain yield (g); PW, Panicle weight (g); DSY, Dry stalk yield (g); FSY, Fresh stalk yield (g); LN, Leaf number; NT, Number of tillers; HI, Harvest index; TW, Test weight (100 seed weight); GN%, N content in grain, SN%, N content in stalk.* * denotes p = 0.05, ** denotes p = 0.001.

### Population Structure and Diversity Analysis

**FIGURE 2:**
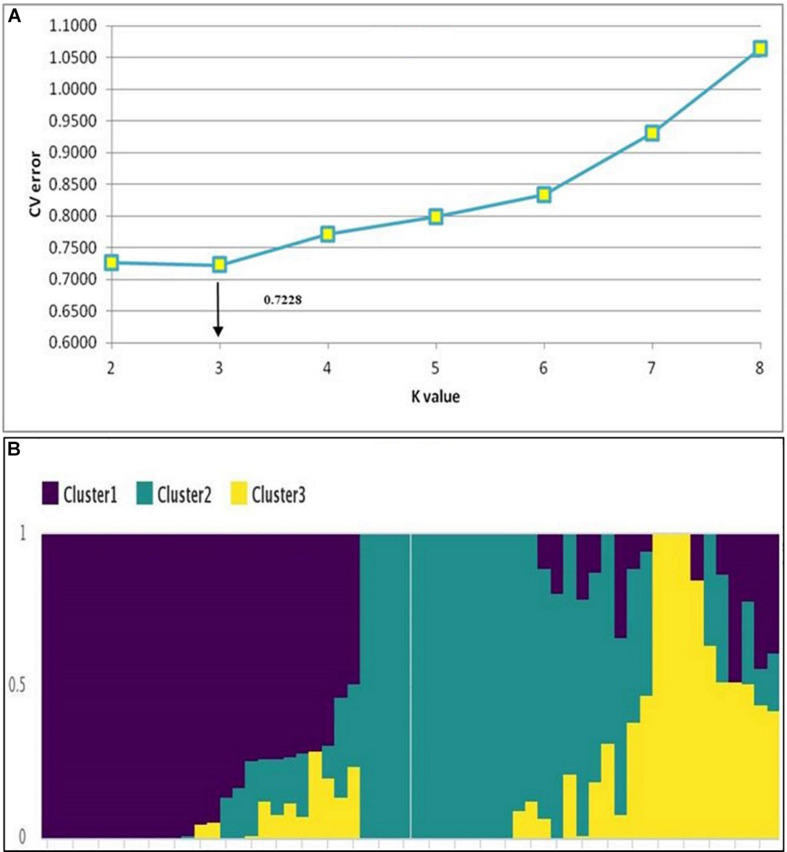
Population structure analysis. **(A)** Rate of change in CV error between successive *K*-values; K-values ranged from 1 to 8. **(B)** Population structure of the 58 sorghum accessions showing three major clusters.

**FIGURE 3:**
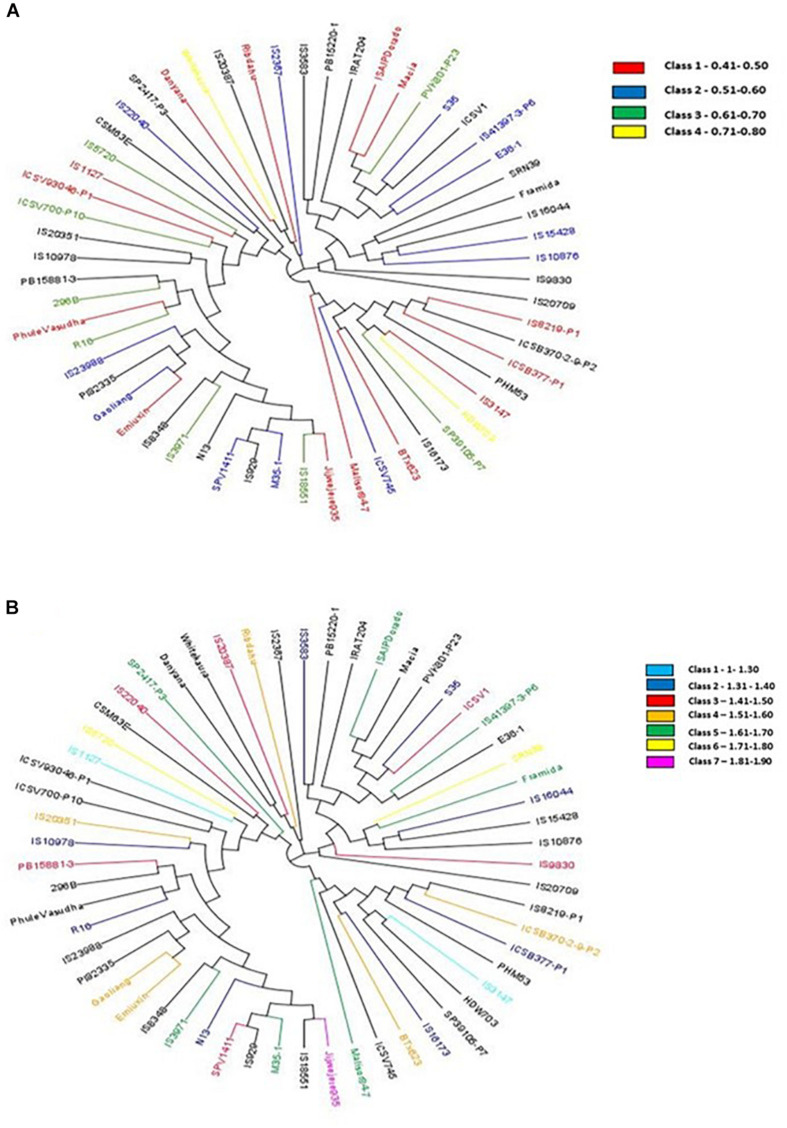
**(A)** Phylogentic tree showing genotypes in different classes based on stalk N (SN%) content [from the range of low (0.41–0.50) to high (0.71–0.80)]. **(B)** Phylogentic tree showing genotypes in different classes based on grain N (GN%) content [from a range of low (1–1.30) to high (1.81–1.90)].

The hierarchical population structure analysis with a range of *k* = 1–8 sub-populations using 89,770 SNPs, helped identify *k* = 3 (Figure 2A) and accessions contributing to each subpopulation (Figure 2B). The percentage of heterozygous alleles is three. Phylogenetic analysis using the GBS data^3^ formed three distinctive clusters (grain N and stalk N data), to develop independent phylogenetic trees. The individuals with high and low N values (for both grain and stalk) were randomly distributed. The largest unit clustered together 13 accessions (in red) for stalk N content ranging from 0.41 to 0.50% followed by a set of 12 accessions (in blue) for the range 0.51–0.60%. For grain N content, the largest cluster consisted of 8 genotypes (in dark blue) with a range of 1.31–1.40%. The other groups 3, 4, and 5 had six genotypes each (in red, orange, and green) with a extensive range of 1.41–1.60% (Figures 3A,B).

### Identification of Contrasting Sorghum Genotypes for NUE

**FIGURE 4:**
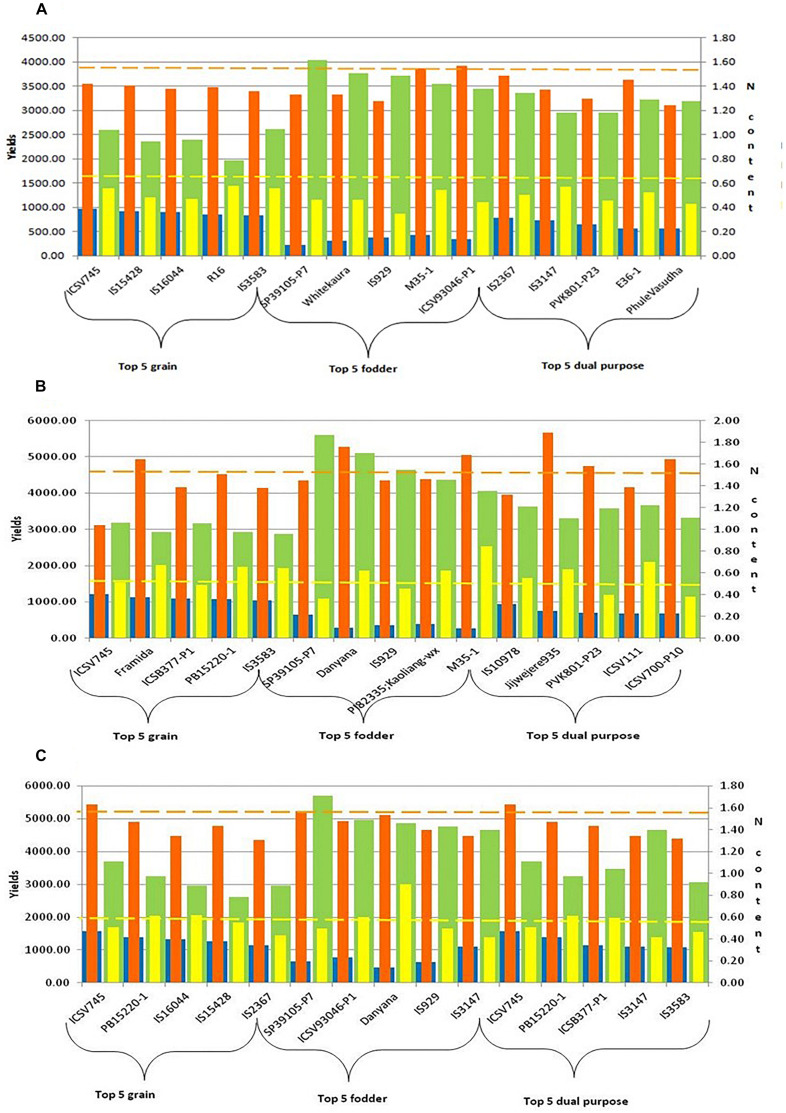
Top five grain, fodder and dual purpose sorghum genotypes under different N (**A:** N0, **B:** N50, and **C:** N100) regimes [Note: 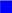 GY (g): Grain yield (g);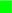 DSY (g): Dry stak yield (g); 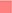 GN%: N content in grain; 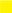 SN%: N content in stak].

A set of 10 accessions each, better and poor performers, was selected based on grain yield data (over two seasons) under low N conditions. Genotypes with high grain yield under low N conditions considered as high NUE genotypes (ICSV745, IS15428, IS16044, R16, IS3583, Gaoliang, IS2367, IS22040, ICSB377-P1, PVK801), and the genotypes with poor performance under low N conditions considered as low NUE genotypes (BTx623, Malisor84-7, PB15881-3, HDW703, IS20709, SP2417-P3, SP39105-P7, Danyana, 296B, and SPV1411). Along with contrasting genotypes, we have also identified the top five product-specific (grain, fodder, and dual-purpose) genotypes under N0, N50, and N100 conditions. These genotypes were identified based on their yield performance in terms of grain, fodder, and both grain and fodder (Figure 4).

### Contrasting Response of NUE and Associated Genes in High and Low NUE Genotypes

Expression analysis of key genes associated with N uptake, assimilation, and remobilization under different N conditions, helps to understand the contrasting response of genes in different tissue samples of high (PVK801) and low (HDW703) NUE genotypes.

#### Ammonium Transporters

The expression profiles of the *SbAMT* genes (Supplementary Table 3) in shoot tissues, under N0 compared with N100 condition, were down-regulated for both test accessions. In shoot samples of PVK801, a down-regulation of 30, 31, 2, and 65-folds, whereas in HDW703 11, 7, 14, and threefold down-regulation was recorded for *SbAMT1-1, SbAMT1-2, SbAMT2-1*, and *SbAMT2-2* genes, respectively.

**FIGURE 5:**
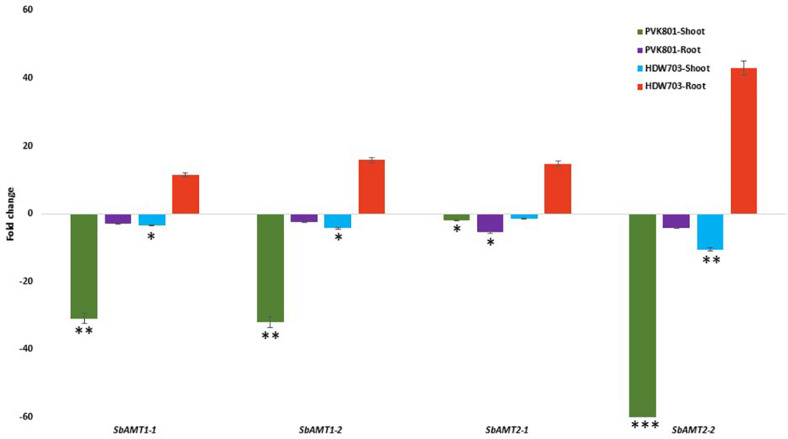
Expression profiles of *SbAMT* genes in shoot and root samples of high (PVK801) and low NUE (HDW703) genotypes, under N0 condition. Here N0 condition taken as treated and N100 used as control. *, **, and *** denotes significance at 5, 1, and 0.1% P, remaining not significant.

However, the root tissues recorded contrasting regulation than shoot tissues. Root samples of PVK801 exhibited 3, 2, 5, and 4-fold down-regulation of *SbAMT1-1, SbAMT1-2, SbAMT2-1*, and *SbAMT2-2*genes, respectively; whereas low NUE genotype HDW703 observed up-regulation of *SbAMT1-1, SbAMT1-2, SbAMT2-1*, and *SbAMT2-2* genes by 11, 15, 14, and 40-folds, respectively (Figure 5).

#### Nitrate Transporters

Similar to ammonium transporter genes shoot samples of high NUE genotype PVK801 showed 12, 66, 32, and 13-folds down-regulation. In contrast, low NUE genotype HDW703 exhibited about 2, 10, 4, and 10-folds down-regulation for *SbNRT1-1A, SbNRT1-1B, SbNRT1-2*, and *SbNRT2-1* genes, respectively in N0 compared to N100 condition.

**FIGURE 6:**
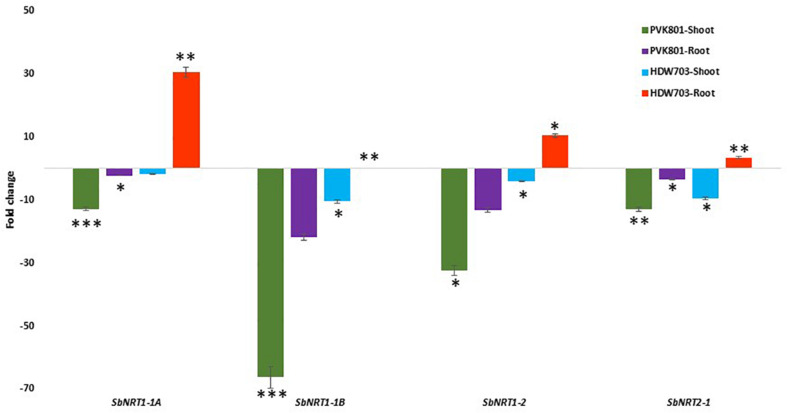
Expression profiles of *SbNRT* genes in shoot and root samples of high (PVK801) and low NUE (HDW703) genotypes, under N0 condition. Here N0 condition taken as treated and N100 used as control. *, **, and *** denotes significance at 5, 1, and 0.1% P, remaining not significant.

*SbNRT* genes exhibited contrasting expression profiles in root samples of high and low NUE genotypes. In high NUE genotype PVK801 about 2, 21, 13, and 3-fold down-regulation was recorded, for*SbNRT1-1A, SbNRT1-1B, SbNRT1-2*, and *SbNRT2-1* genes, respectively. On the contrary, in HDW703 about 30, 2, 10, and 4-fold up-regulation of *SbNRT1-1A, SbNRT1-1B, SbNRT1-2*, and *SbNRT2-1* genes was observed (Figure 6).

#### N Assimilation and Remobilization Related Genes

In shoot samples of high NUE genotype PVK801, N assimilatory and remobilization related genes *SbNiR[NADH]3, SbGS, SbGOGAT[NADH], SbGOGAT[Fd]*, and *SbGDH* down-regulated by 30, 7, 59, 38, and 3-folds, respectively. For low NUE genotype HDW703 exhibited 11, 7, 14, and 3-fold down-regulation of *SbNiR[NADH]3, SbGS, SbGOGAT[NADH]*, and *SbGOGAT[Fd]* genes respectively. On the contrary to other genes, *SbGDH* exhibited about twofold up-regulation in shoot samples of HDW703 in N0 compared to the N100 condition.

**FIGURE 7:**
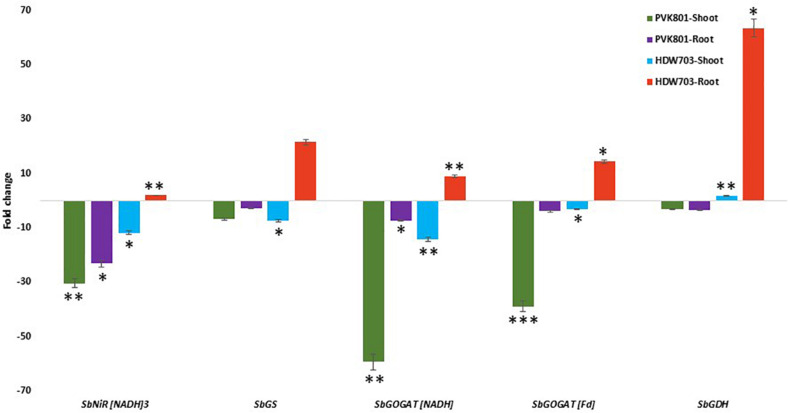
Expression profiles of assimilatory and remobilization related genes in shoot and root samples of high (PVK801) and low NUE (HDW703) genotypes, under N0 condition. Here N0 condition taken as treated and N100 used as control. *, **, and *** denotes significance at 5, 1, and 0.1% P, remaining not significant.

Root samples of PVK801 exhibited down-regulation of all the above genes in the N0 condition. About 23, 3, 7, 4, and 4-fold down-regulation of *SbNiR[NADH]3, SbGS, SbGOGAT[NADH], SbGOGAT[Fd]*, and *SbGDH* genes in N0 compared to N100 condition was observed. However, contrasting expression of N assimilatory and remobilization related genes in low NUE genotype (HDW703), with about 2, 21, 9, 14, and 63-fold up-regulation of *SbNiR[NADH]3, SbGS, SbGOGAT[NADH], SbGOGAT[Fd]*, and *SbGDH* genes, respectively, were noted (Figure 7).

## Discussion

We conducted a series of field and lab experiments to understand the genotype response, nitrogen dosage effects, and season-specific variations associated with NUE in a set of sixty diverse sorghum accessions. Different physiological, agronomical, and biological yield attributes associated with NUE were systematically studied in three different N dosages (0, 50, and 100% of the recommended N) over two seasons.

With the increase in N dose, the higher SPAD readings were observed, This variation could be of increased availability of N in leafs under N50 and N100 conditions, similar variations were reported in three sorghum varieties of Nigeria (Ajeigbe et al., 2018) and rice (Hassan et al., 2009), wheat (Noulas et al., 2018). This response was also similar to dicotyledons plants (Poorter and Evans, 1998), spinach (Gülser, 2005). An increase in plant height with an increased nitrogen rate application, which might be attributed to effect of N application in plant growth and development. Our results are in accordance with studies in sorghum varieties of east Africa and Nigeria (Shamme and Raghavaiah, 2016; Ajeigbe et al., 2018) and rapeseed (Khan et al., 2017). Generally physiological maturity of sorghum accelerated under low N than high N conditions, interestingly in this study flowering time showed a minimum response to N doses at field in accordance to few sorghum varieties of Nigeria (Ajeigbe et al., 2018), mung bean (Achakzai et al., 2012) and wheat (Guttieri et al., 2017). Tiller number of the present study increased with enhanced N rates. It is evident that, optimum N availability stimulates tiller number, hence increased number of panicles there by yield improvement. Shamme and Raghavaiah. (2016) and Ajeigbe et al. (2018) also reported higher number of tillers and panicles and weight of panicles with increasing N application in sorghum, in *Brachypodium* (Yang and Udvardi, 2018) and in mung bean (Achakzai et al., 2012) and rice (Pan et al., 2016). There have been reports on N promoting the spikelet number per panicle and thus yield (Yoseftabar, 2013). The analysis of variance revealed significant influence of genotypes, N dosage treatments and their interaction on grain yield which is in agreement with the earlier reports (Gelli et al., 2016; Shamme and Raghavaiah, 2016). Grain yield of sorghum is the final outcome of yield components, more than 40% reduction in grain yield was observed in N0 compared to N100 for individual and across-season of field evaluations. Significant increase in grain yield with the increased N fertilizer application was well established, as N is the main macro nutrient critical for increased yield and related components such as panicle numbers and tiller numbers in major cereals (Gallais and Hirel, 2004; Laperche et al., 2006; Gelli et al., 2016; Shamme and Raghavaiah, 2016; Srikanth et al., 2016). Less than 8% decrease in test weight was observed amongst genotypes grown in N0 compared to N100 conditions in both the seasons, which could be because of increased spikelet number with increase in N application may affect the spikelet size (Qiao et al., 2013). Contrastingly, it was also reported that, test weight is an important trait and yield determining component, reported to be a genetic and also a stable varietal trait mostly depends on spikelet size hence not influenced by environmental factors (Ashraf et al., 1999; Kanfany et al., 2014).

Harvest index is the measure of success in translocation of absorbed assimilates (from source) into economic yields (to sink). Genotypes with more harvest index distribute carbohydrate in to the product efficiently (Li et al., 2012). In the present study increase in the N dosages consistently improved the harvest index, it was significant within genotypes, season and interaction effects of genotypes with seasons indicates the existence of substantial genetic variability for this trait and could be used as selection criterion for improvement of NUE. About 26% reduction in harvest index was observed in N0 conditions in comparison with recommended N (N100). Our results are in line with the earlier studies explaining harvest index increased with the increase in fertilizer N supply in sorghum and other crops (Lawrence et al., 2008; Shamme and Raghavaiah, 2016; He et al., 2017).

Significant variations for grain N content was observed within genotypes and the seasons. Grain N content was gradually increased by increasing fertilizer N applications. About 9 and 17% reduction in grain N content was observed in the present study suggesting the complexity of this trait in different treatments. N content in stover exhibited significant variations in genotypes, treatments, seasons, and their interactions. N content in grain and stover and its association with grain yield and biomass appears to be highly variable across genotypes, seasons, locations, crop duration of genotypes and timing of N application (Zhang et al., 2009; Artacho et al., 2011; Gueye and Becker, 2011; Zhao et al., 2012; Sui et al., 2013; Li et al., 2014; Gelli et al., 2016; Shamme and Raghavaiah, 2016; He et al., 2017).

### Correlation Coefficient Analysis

In all the three N treatments, correlation coefficient analysis revealed significant correlations for grain yield, which is positively correlated with panicle weight and harvest index, except across seasons. In a recent study on rapeseed similar correlation trends were observed where genotypes differed significantly in agronomic traits with no consistency in correlation among morphological and N utilization efficiency (He et al., 2017). Interestingly, significant negative correlations were observed for fresh stover weight, dry stover weight, leaf area with N content in grain. Contrary to our results, there are reports in sorghum and other crops describing, grain yield strongly correlated with biomass yield suggesting the role of economic sink strength (Ntanos and Koutroubas, 2002; Gelli et al., 2016; Srikanth et al., 2016). This could be attributed to the differential response of photo-sensitivity and dual-purpose nature of most of the accessions involved in this study. Correlation of the yield and other associated NUE traits appear to be variable with N fertilizer dosage and the genotypes and/or varieties (Sui et al., 2013; Zhang et al., 2013). In the present study, N content in grain positively correlated with panicle weight, grain yield and harvest index under N0 conditions, suggests these traits importance in screening high NUE lines of sorghum. Fresh and dry stover yield positively correlated with leaf area, plant height, days to 50% flowering in all the treatments and all the conditions. Leaf area correlated with leaf number, dry stover yield in all the treatments of the field trials. N uptake, assimilation and its distribution in vegetative, and reproductive parts are critical processes which determine the grain yield (Guindo et al., 1992). Panicle number significantly correlated with leaf number and number of tillers in all the treatments of the present field study proposes the differential responses of yield and associated traits and their role in plant growth and development under differential nitrogen conditions in sorghum.

### Population Structure and Diversity Studies

The sorghum genotypes selected for the study represent the genetic diversity of different countries viz., India, Niger, Sudan, South Africa, Pakistan, Yemen, Cameroon, and the United States depicting the range of genotypes and their origin. The phylogenetic tree from the GBS data formed three distinctive clusters. The high diversity of plant populations may be due to the evolutionary history, genetic drift, and geographic range of the species and their characteristics (Al Salameen et al., 2020). The genotypes representing the grain N and stalk N were randomly distributed over the phylogenetic tree. A similar trend of high genetic variation was observed in sorghum landraces and 44 genotypes selected at random from sorghum mini-core collection (Dossou-Aminon et al., 2015; Satish et al., 2016). Similar overlapping of genotypes in phylogenetic tree was observed in *RhanteriumeppaposumOliv*. (Arfaj). The major reason was due to low genetic distance and differentiation among the populations (Hogbin and Peakall, 1999).

### Identification of Contrasting Sorghum Genotypes Under Varying N Conditions

Sorghum is grown across the world for food-fodder-feed-fuel purpose. This is also include industrial use of silage and biofuel applications, especially in developed world. These different end uses are delivered through breeding products developed for specific purpose and with specific cultivation practices in a given agro-ecology. This also include differential N application and N use scenarios. Accelerating the sorghum productivity in terms of grain and fodder yield under intensive agriculture with high dose of N fertilizer application is a cost effective approach for industrial needs. Similarly improving N use efficiency under substance farming (with no or minimal fertilizer inputs) for poor and marginal farmers is possible and also help realizing climate resilience. In this study, apart from product specific (grain, fodder and dual purpose) sorghum accessions (Figure 4) in varying N conditions, a set of better and poor performing accessions specifically under low N conditions were identified to investigate the molecular basis for low N tolerance in sorghum. Identification of contrasting (high and low NUE) accessions is the first step toward deciphering the candidate genes and pathways associated with N metabolism, addressing to the different end-uses of sorghum cultivars.

### Differential Expression of NUE Associated Genes in Contrasting Sorghum Genotypes Under Varying N Regimes

Using quantitative real-time PCR analysis, expression profiles of N uptake, assimilation and remobilization related genes were studied in shoot and root samples of high and low NUE sorghum genotypes. In these assays N0 condition taken as treated and N100 (recommended N) condition considered as control. Ammonium and nitrate are the major sources of fertilizer N in agricultural soils and act as signal and nutrients for plant growth and development. N fertilizer source nitrate is usually absorbed by plant roots by *NRT1* and *NRT2* (low and high-affinity nitrate transporters), which is further reduced to nitrite by nitrate reductase gene (NR), and converted to ammonium by nitrite reductase (NiR). Ammonium further assimilated into amino acids by glutamine synthetase (GS) and glutamate synthase (GOGAT) genes (Crawford, 1995; Lam et al., 1996; Stitt, 1999).

Expression analysis of NUE associated genes under varying N doses in different crops has exhibited differential expression profiles in different tissue samples. Ammonium is one of the readily available form of N for the plants and NH4^+^ uptake in plant roots and its transport to shoots is mediated by ammonium transporters belonging to the *AMT* family. In crop plants, *AMT1*, *NRT2* along with the support of *NRT3* genes act as high-affinity transporters (HATs) in low N situations (Masclaux-Daubresse et al., 2010; Zhou et al., 2016). On the other hand, *AMT2* and *NRT1* works as low affinity transporters (LATs) performs competently under high dose of ammonium and nitrate (Neuhäuser et al., 2007; Wang et al., 2012). HATs and LATs exhibit differential affinities toward N source *viz*., ammonium and nitrate, it is expected that crops can use a wide range of soil N source applications. In the present study, root samples of low NUE genotype HDW703 exhibited up-regulation of all the *SbAMT* genes (*SbAMT1-1, SbAMT1-2, SbAMT2-1*, and *SbAMT2-2*) in N0 compared to N100 situation. On the contrary root samples of high NUE genotype PVK801 exhibited down-regulation of all the *SbAMT* genes. In rice (Sonoda et al., 2003), also reported similar results, a constitutive expression profiles of *OsAMT1-1* gene well known to be an important member of HAT sub-family, was reported in shoot and root samples. It was well documented that, *AMT1-1* is one of the key gene and potential candidate for improving NUE, plant growth, and grain yield for both low and optimal fertilizer N situations (Ranathunge et al., 2014).

In shoot samples of PVK801 and HDW703 down-regulation of all the *SbNRT* genes (*SbNRT1-1A, SbNRT1-1B, SbNRT1-2*, and *SbNRT2-1*) was observed in N0 condition compared to N100. In root samples of high NUE genotype PVK801 all the *SbNRT* genes of the study showed down-regulation in N0 conditions whereas low NUE genotype HDW703 exhibited up-regulation of all the *SbNRT* genes under N0 conditions. Contrasting expression profiles of these genes in high and low NUE genotypes indicate different genetic background effects (including breeding and selection history). This clearly reflects in *NRT* genes responses toward nitrate dosages. Variations in the expression of the *SbNRT* genes in sorghum leaves and roots between N0 and N100 conditions might be varied with the quantitative fold changes in gene copies also. Enhanced expression of *OsAMT1-1* and *OsNRT2-1* gene was reported in rice seedlings grown in hydroponics conditions using growth media supplemented with low N (Shi et al., 2010). In the roots of sorghum and maize seedlings, *SbNRT1-1A* and *SbNRT1.1B* genes exhibited enhanced expression under N limited conditions (Matsumura et al., 1997; Awada, 2017). Our results are in agreement with the previous study by Fan et al. (2007), Nitrate reductase activity reduced in N sensitive genotype but not altered in NUE rice cultivar under limited fertilizer N condition in hydroponics. One probable reason for the enhanced expression of *NRT* genes in low NUE genotype HDW703 could be increased nitrate accumulation in roots. Another probability could be these *NRT* genes capability to act as potential N sensors that facilitate plants to sense and exploit available nitrate source in soil. It is well characterized that variation for improved N absorption (putatively by *NRT* genes) under low N is contributing to variation in NUE (Masclaux-Daubresse et al., 2010; Shi et al., 2010; Awada, 2017).

It was well established that, N source as a signal induces the differential expression of NUE associated genes including *NRT1*, *NRT2*, *NR*, *NiR*, *GS*, and *GOGAT* (Campbell et al., 1986; Crawford, 1995; Lam et al., 1996; Stitt, 1999). Similar trends were found in our studies with shoot samples of high NUE genotype PVK801 exhibiting down-regulation of *SbNiR[NADH]3, SbGS, SbGOGAT[NADH], SbGOGAT[Fd]*, and *SbGDH* genes. Root samples of PVK801 exhibited down-regulation of all the above genes in N0 condition. Low NUE genotype HDW703 exhibited down- regulation of N assimilatory and remobilization related genes (*SbNiR[NADH]3, SbGS, SbGOGAT[NADH]*, and *SbGOGAT[Fd]*) in shoot samples except *SbGDH* genes showed up-regulation. Whereas in root samples of HDW703 all the assimilatory and remobilization associated genes (*SbNiR[NADH]3, SbGS, SbGOGAT[NADH], SbGOGAT[Fd]*, and *SbGDH)* up-regulated in N0 compared to N100. Differential expression of NiR and GS between low and high NUE genotypes indicated these enzymes active involvement in imparting efficient uptake and utilization of fertilizer N (Hakeem et al., 2012). Activities of *OsGS, OsGOGAT*, and *OsGDH* in a rice hybrid under five N doses described the role of ammonium assimilation enzymes in grain yield improvement and also NUE (Sun et al., 2012). In rice seedlings, Hirose et al. (1997) demonstrated enhanced expression of *NADH-GOGAT* by inducing with NH_4_Cl in root tissues. Sonoda et al. (2003) also got similar results, mRNA accumulation of *NADH-GOGAT* remarkably increased of ammonium induction (after 60 min), while the expression pattern of cytosolic *GS1* (Sakamoto et al., 1989) was constitutive throughout the ammonium induction (Tobin and Yamaya, 2001). Another study in barley demonstrated the expression of GS and GDH during early seed development stage (Hansen et al., 2009). Grabowska et al. (2012) reported, differential expression patterns of*TsGS1*-*3* and *TsGS2*-*1* (low),*TsGDH1*(high) for compensating the low expression levels of *GS* genes in seeds. Similar conclusions were also drawn by Tabuchi et al. (2005). Enhanced expression of these genes in root tissues of low NUE genotype HDW703 of our study explains the role of these genes in nitrogen assimilation.

While crop responsiveness to nitrogen availability depends on both crop variety/genotype and it’s interaction with the level of N fertilization (Chardon et al., 2010), the possible solutions for- continued improvement in crop productivity under intensive production system and sustainable crop performance under marginal, resource-poor systems will require optimization of genetic, management and policy interventions. It is thus clear that the different sorghum production ecologies, high N-input (intensive) vs., low/no-input, will need differential genetic (and management) solutions for NUE. This will include appropriate gene constellation and deployment of specific genetic donors through targeted breeding efforts. Additional layer of complexity of crop utilization, *viz*., grain, forage, or dual-purpose, also require specific inputs in terms of crop modulation and breeding for a specific product. We were able to document this variation (Figures 4A–C) by identifying better germplasm sources for grain, forage and dual-purpose products. This information along with putative candidate genes governing different NUE mechanisms will help to advance the NUE research in sorghum, especially for developing better-targeted breeding for improved productivity and livelihoods of the sorghum growing farming communities across the globe.

## Conclusion

In the current climate change scenario and continuous global perusal of food security, improving NUE of sorghum for both intensive production ecologies and subsistence ecologies is one of the major goals in sorghum improvement programs. In the high N fertilizer application scenario most commonly observed in the intensive agricultural systems, variation in NUE is primarily triggered by differences in N uptake capacity. In contrast, under limited N conditions such as in agro-ecologies of sorghum production areas in Asia and Africa, NUE variation is driven by changes in N remobilization and utilization efficiency. The screening and identification of robust traits in a complex field evaluation study over two seasons is the first of its kind in NUE research and/or nutrition research. In the present study, different leaf traits, growth traits, panicle and biological yield traits along with NUE traits of 60 diverse sorghum genotypes were systematically evaluated at three N dosages at field level over two seasons. Significant variations were observed for different yield and yield-related traits in 0 and 50% N regimes confirming the availability of genotypic variability in sorghum under low N conditions. There is continuous reduction in the composite yield parameters with the reduction in N application. Correlation coefficient analysis revealed the importance of panicle weight and harvest index in different N environments, and these two traits may be ideal traits for identifying N use efficient genotypes. Contrasting sorghum genotypes identified in this study could be crucial to spot genes for NUE and associated traits. Expression profiling of NUE related genes in shoot and root tissues of contrasting lines (PVK801 and HDW703) raised in varying N conditions (N0 vs., N100) revealed some interesting outcomes. Root tissues of contrasting lines exhibited differential expression profiles for uptake (*SbAMT* and *SbNRT*), assimilation (*SbGS*, *SbGOGAT[NADH], SbGOGAT[Fd]*, and *SbNiR[NADH]3)*, and amino acid biosynthesis associated genes (*SbGDH)*. This study identified some of the promising sorghum genotypes with buffer capacity for yield under low N conditions. This study also identified better performing grain, stover and dual-purpose sorghum genotypes. These genotypes could be potential candidates for in-depth molecular analysis and deployment into the crop improvement programs to develop high NUE sorghum cultivars.

## Data Availability Statement

The raw data supporting the conclusions of this article will be made available by the authors, without undue reservation.

## Author Contributions

RG led the project and developed the framework. RG and SD conceptualized and designed this research strategy. SD contributed to the experimental design, genetic material selection, and genomics studies. GC designed the N-dose treatments and N-response observations. SB, KR, and LR performed field and lab experiments, phenotyping, analyzed, and interpreted the data. AR, AV, RD, and PG carried out statistical and bioinformatics analysis. SB, LR, SD, and RG wrote the manuscript. All authors contributed to editing the MS for publication.

## Conflict of Interest

The authors declare that the research was conducted in the absence of any commercial or financial relationships that could be construed as a potential conflict of interest.

## Footnotes

1 https://doi.org/10.21421/D2/WCAHRK

2 http://simgene.com/Primer3

3 https://doi.org/10.21421/D2/BYZY2F

## Supplementary Material

Supplementary Figure 1 Weather data of field during the years 2016–17 for the complete crop growth cycle.

Supplementary Figure 2 Weather data of field during the years 2017–18 for the complete crop growth cycle.

Supplementary Table 1 List of sorghum genotypes used for NUE field phenotyping experiment.

Supplementary Table 2 Details of soil properties of the NUE field experiment for the year 2016 at ICRISAT, Patancheru.

Supplementary Table 3 List of gene specific primers used for studying expression profiles of N uptake assimilatory and remobilization related genes in shoot and root tissues of contrasting sorghum genotypes.

Supplementary Table 4 Correlation coefficient analysis of 60 genotypes at N0 **(A)**, N50 **(B)**, and N100 **(C)** dosages for 2016–2017 at ICRISAT, Patancheru.

Supplementary Table 5 Correlation coefficient analysis of 60 genotypes at N0 **(A)**, N50 **(B)**, and N100 **(C)** dosages for 2017–2018 at ICRISAT, Patancheru.
