# Supplementary figures and images for: Nitrogen Use Efficiency in Sorghum: Exploring Native Variability for Traits Under Variable N-Regimes
Source: Front Plant Sci. 2021 Apr 21;12:643192. doi: 10.3389/fpls.2021.643192 (PMC8097177; doi:10.3389/fpls.2021.643192)

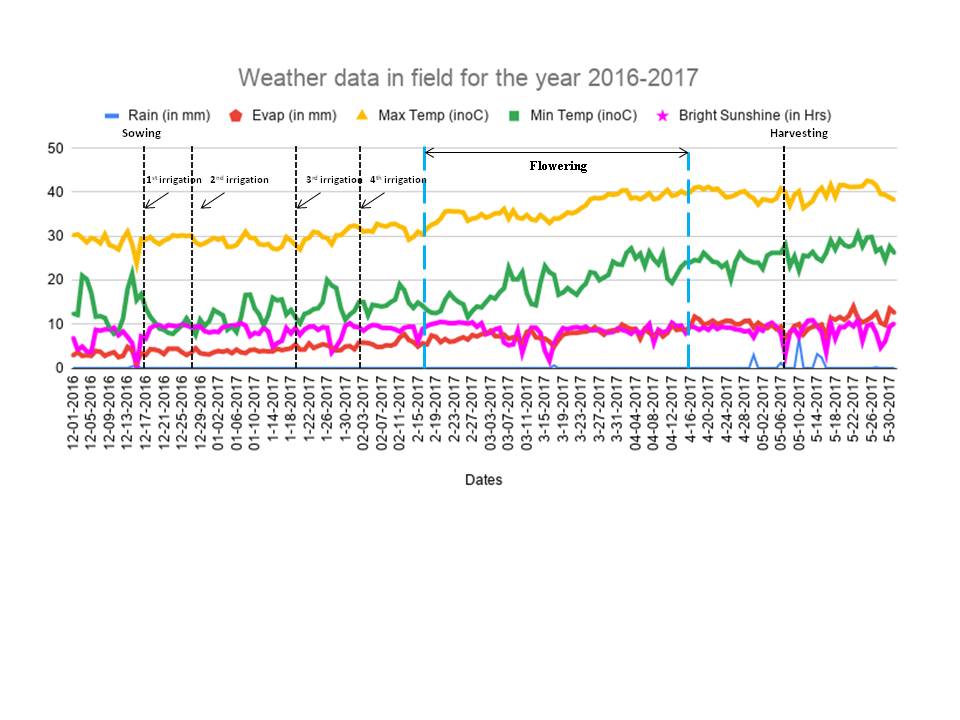

Supplement: Supplementary Figure 1 — Weather data of field during the years 2016–17 for the complete crop growth cycle. [file Image_1.JPEG]

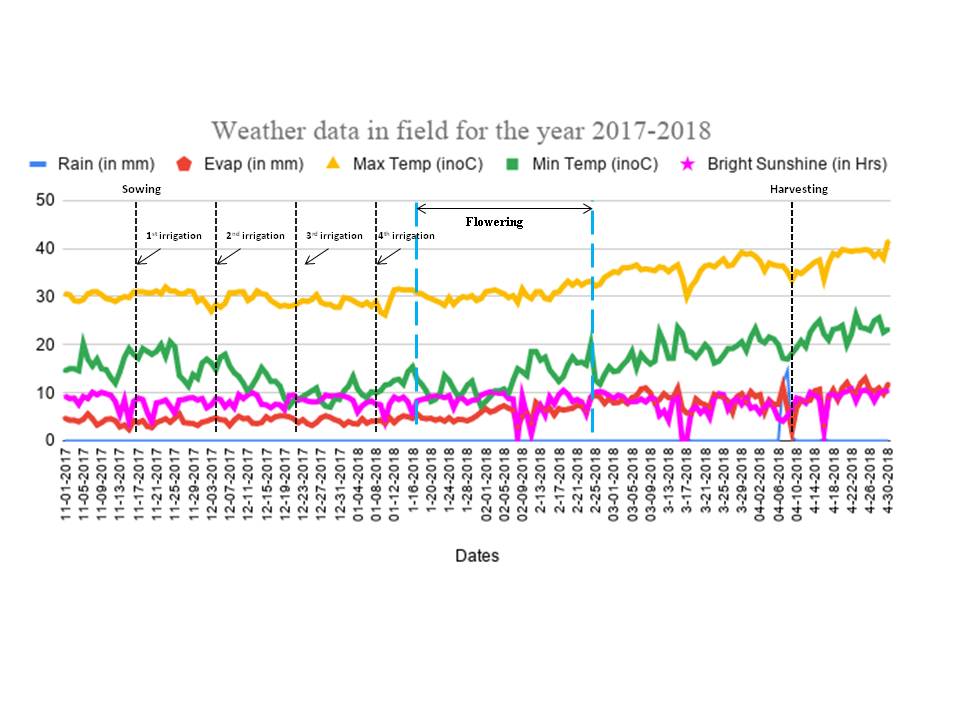

Supplement: Supplementary Figure 2 — Weather data of field during the years 2017–18 for the complete crop growth cycle. [file Image_2.JPEG]
