# Supplementary material for: Nitrogen Use Efficiency in Sorghum: Exploring Native Variability for Traits Under Variable N-Regimes
Source: Front Plant Sci. 2021 Apr 21;12:643192. doi: 10.3389/fpls.2021.643192 (PMC8097177; doi:10.3389/fpls.2021.643192)
Supplement: Supplementary Table 1 — List of sorghum genotypes used for NUE field phenotyping experiment. [file Table_1.DOCX]

**Supplementary table 1.** List of sorghum genotypes used for NUE field phenotyping experiment

| **S.no** | **Genotypes** |
| --- | --- |
| 1. | IS929 |
| 2. | IS1127 |
| 3. | IS2367 |
| 4. | IS3147 |
| 5. | IS3583 |
| 6. | IS3971 |
| 7. | IS5720 |
| 8. | IS8348 |
| 9. | IS10876 |
| 10. | IS10978 |
| 11. | IS15428 |
| 12. | IS16044 |
| 13. | IS16173 |
| 14. | IS20351 |
| 15. | IS20387 |
| 16. | IS20709 |
| 17. | IS22040 |
| 18. | IS23988 |
| 19. | BTx623 |
| 20. | IS18551 |
| 21. | E36-1 |
| 22. | IS9830 |
| 23. | ICSV745 |
| 24. | S35 |
| 25. | M35-1 |
| 26. | Macia |
| 27. | CSM63E |
| 28. | Ribdahu |
| 29. | R16 |
| 30. | IRAT204 |
| 31. | SPV1411(Parbani moti) |
| 32. | 296B |
| 33. | ICSV1 |
| 34. | ICSB377-P1 |
| 35. | ICSB370-2-9-P2 |
| 36. | N13(EC861959) |
| 37. | SP2417_P3 |
| 38. | IS41397_3_P6 |
| 39. | ICSV93046-P1 |
| 40. | ICSV700-P10 |
| 41. | Phule_Vasudha |
| 42. | ICSV111 |
| 43. | ISAIPDorado |
| 44. | PB15220-1 |
| 45. | PVK801-P23 |
| 46. | BulkY-P1 |
| 47. | SRN39(EC861960) |
| 48. | Whitekaura(EC861950) |
| 49. | Danyana(EC861945) |
| 50. | Framida(EC861955) |
| 51. | Malisor84-7 (EC861952) |
| 52. | Erniuxin(IS30318) |
| 53. | HDW703(IS20624) |
| 54. | PI82335;Kaoliang-wx (IS3771) |
| 55. | PHM53(IS29409) |
| 56. | Jijwejere935(IS18551) |
| 57. | Gaoliang(IS30405) |
| 58. | PB15881-3(ICSV745) |
| 59. | IS8219-P1 (ICSB370) |
| 60. | SP39105-P7 (ICSR93024) |
