# Supplementary material for: Nitrogen Use Efficiency in Sorghum: Exploring Native Variability for Traits Under Variable N-Regimes
Source: Front Plant Sci. 2021 Apr 21;12:643192. doi: 10.3389/fpls.2021.643192 (PMC8097177; doi:10.3389/fpls.2021.643192)
Supplement: Supplementary Table 2 — Details of soil properties of the NUE field experiment for the year 2016 at ICRISAT, Patancheru. [file Table_2.DOCX]

**Supplementary table 2**: Details of soil properties of the NUE field experiment for the year 2016 at ICRISAT, Patancheru

|  | | **S.NO** | **S.Id** | **pH** | **EC** | **OC** | **Avail-P** | **Exch-K** | **Exch-Ca** | **Exch-Mg** | **Avail-S** | **Avail-Zn** | **Avail-B** | **Avail-Fe** | **Avail-Cu** | **Avail-Mn** | **Exch-Na** |
| --- | --- | --- | --- | --- | --- | --- | --- | --- | --- | --- | --- | --- | --- | --- | --- | --- | --- |
|  | |  |  |  | **dS/m** | **%** | **ppm** | **ppm** | **ppm** | **ppm** | **ppm** | **ppm** | **ppm** | **ppm** | **ppm** | **ppm** | **ppm** |
|  | |  |  |  |  |  |  |  |  |  |  |  |  |  |  |  |  |
| **BP-12 field soil Samples** | | 1 | BP 12 | 7.62 | 0.09 | 0.33 | 6.73 | 150 | 3985 | 607 | 3.55 | 0.83 | 0.94 | 9.28 | 1.13 | 6.83 | 354 |
|  |  | 2 | BP 12 | 7.58 | 0.08 | 0.32 | 6.68 | 152 | 4025 | 615 | 3.66 | 0.82 | 0.96 | 9.12 | 1.12 | 6.54 | 311 |
|  |  | 3 | BP 12 | 7.60 | 0.10 | 0.34 | 6.73 | 149 | 4003 | 608 | 3.54 | 0.81 | 0.93 | 9.38 | 1.12 | 6.67 | 294 |
|  | |  | **Mean** |  |  | **0.33** | **6.71** | **150** | **4004** | **610** | **3.59** | **0.82** | **0.94** | **9.26** | **1.12** | **6.68** | **320** |
|  | |  | **Remarks** | **Normal** | **Normal** | **Low** | **Medium** | **high** | **High** |  | **Deficient** | **Sufficient** | **Sufficient** | **Sufficient** | **Sufficient** | **Sufficient** |  |
|  |  |  |  |  |  |  |  |  |  |  |  |  |  |  |  |  |  |
|  | |  |  |  |  | *Low soil C levels indicate low available nitrogen | | | | |  |  |  |  |  |  |  |
