# Supplementary material for: Nitrogen Use Efficiency in Sorghum: Exploring Native Variability for Traits Under Variable N-Regimes
Source: Front Plant Sci. 2021 Apr 21;12:643192. doi: 10.3389/fpls.2021.643192 (PMC8097177; doi:10.3389/fpls.2021.643192)
Supplement: Supplementary Table 3 — List of gene specific primers used for studying expression profiles of N uptake assimilatory and remobilization related genes in shoot and root tissues of contrasting sorghum genotypes. [file Table_3.DOCX]

**Supplementary table 3**: List of gene specific primers used for studying expression profiles of N uptake assimilatory and remobilization related genes in shoot and root tissues of contrasting sorghum genotypes

| **S. No.** | ***Gene name*** | **Primer sequences forward 5'-3'** | **Primer sequences reverse 5'-3'** | **Tm ^o^C** |
| --- | --- | --- | --- | --- |
| 1 | *SbAMT1-1* | CGGCTTCGACTACGACTTCT | ATGAGGTAGGCGACGAACTG | 60 |
| 2 | *SbAMT1-2* | CGGCTTCGACTACGACTTCT | GCGGAGTAGATGAGGTACGC | 60 |
| 3 | *SbAMT2-1* | CGGATACGTCATCCACCTCT | CATCAGCAGGATGTTGTTCG | 60 |
| 4 | *SbAMT2-2* | CGGATACGTCATCCACCTCT | CCCTGTCGCTCTTCAGTCTC | 60 |
| 5 | *SbNRT1.1A* | GACGTGGAGGAGGTGAAGAC | GACACCGAGAAGGTGGTCAT | 60 |
| 6 | *SbNRT1.1B* | CTGACCCGGACTTGCTCTAC | GAACCTGCATTCCTTGCTGT | 60 |
| 7 | *SbNRT1.2* | CCCATCTTCTTGTCCACCAT | GCGAGGATGATGATGAAGGT | 60 |
| 8 | *SbNRT2.1* | TCCATCTTCTCCCGTCTCAC | GAGGAACCTGACGACGATGT | 60 |
| 9 | *SbNiR[NADH] 3* | ACAATGACTTCCTGCCCATC | ACCTCAACTCGCGTCACTTT | 60 |
| 10 | *SbGS* | CCGGAGTACACCAAGGACAT | GTTCACGAGCCTTGAGGAAG | 60 |
| 11 | *SbGOGAT [NADH]* | TGCTTGAGAGAATGGCACAC | AACCCAGCATCCTTTGTGAC |    \| 60 \| \| --- \| |
| 12 | *SbGOGAT[Fd]* | ACCAACTCGTCCTGCCATAC | CTGAGCCGTATCAGCAACAA | 60 |
| 13 | *SbGDH* | TCCATTCAGGGAGATCAAGG | GTCCCCTAGCGTTGTCATGT | 60 |
| 14 | *SbUbi*  (Internal control) | CAAGGAGTGCCCCAACAC | GGTAGGCGGGTAAAGCAAA | 60 |
