# Supplementary material for: Nitrogen Use Efficiency in Sorghum: Exploring Native Variability for Traits Under Variable N-Regimes
Source: Front Plant Sci. 2021 Apr 21;12:643192. doi: 10.3389/fpls.2021.643192 (PMC8097177; doi:10.3389/fpls.2021.643192)
Supplement: Supplementary Table 4 — Correlation coefficient analysis of 60 genotypes at N0 (A), N50 (B), and N100 (C) dosages for 2016–2017 at ICRISAT, Patancheru. [file Table_4.DOCX]

**Supplementary table 4:** Correlation coefficient analysis of 60 genotypes at N0(4A), N50 (4B) and N100 (4C) dosage for 2016-2017 at ICRISAT, Patancheru.

4A

4B

**4C**

| **Trait** | **DF50** | **AF** | **LA** | **PHT** | **PN** | **GYLDg** | **PWg** | **DSYLDg** | **FSYLDg** | **LN** | **NT** | **HI** | **SW100** | **Grain_N** | **Stalk_N** |
| --- | --- | --- | --- | --- | --- | --- | --- | --- | --- | --- | --- | --- | --- | --- | --- |
| **DF50** | 1 |  |  |  |  |  |  |  |  |  |  |  |  |  |  |
| **AF** | -0.29* | 1 |  |  |  |  |  |  |  |  |  |  |  |  |  |
| **LA** | 0.55** | -0.22 | 1 |  |  |  |  |  |  |  |  |  |  |  |  |
| **PHT** | 0.25 | -0.18 | 0.23 | 1 |  |  |  |  |  |  |  |  |  |  |  |
| **PN** | -0.2 | -0.01 | -0.2 | 0.12 | 1 |  |  |  |  |  |  |  |  |  |  |
| **GYLDg** | 0.01 | 0.03 | -0.07 | -0.1 | -0.09 | 1 |  |  |  |  |  |  |  |  |  |
| **PWg** | 0.15 | 0.02 | 0.02 | -0.13 | -0.15 | 0.83** | 1 |  |  |  |  |  |  |  |  |
| **DSYLDg** | 0.57** | -0.18 | 0.61** | 0.39** | -0.06 | 0.04 | 0.03 | 1 |  |  |  |  |  |  |  |
| **FSYLDg** | 0.49** | -0.19 | 0.62** | 0.39** | 0.07 | 0.02 | 0.05 | 0.86** | 1 |  |  |  |  |  |  |
| **LN** | 0.001 | -0.2 | 0.44** | 0.1 | 0.22 | -0.32* | -0.25 | 0.22 | 0.23 | 1 |  |  |  |  |  |
| **NT** | -0.39** | -0.13 | 0.02 | 0.02 | 0.58** | -0.28* | -0.3* | -0.15 | -0.04 | 0.45** | 1 |  |  |  |  |
| **HI** | -0.36** | 0.13 | -0.33* | -0.29* | -0.12 | 0.78** | 0.6** | -0.5** | -0.42** | -0.38** | -0.18 | 1 |  |  |  |
| **SW100** | -0.2 | 0.19 | -0.13 | -0.13 | -0.47** | 0.07 | 0.15 | -0.08 | -0.2 | -0.07 | -0.21 | 0.13 | 1 |  |  |
| **Grain_N** | 0.11 | 0.03 | 0.2 | -0.16 | 0.002 | -0.11 | -0.09 | -0.06 | 0.12 | -0.21 | 0.01 | 0.004 | -0.14 | 1 |  |
| **Stalk_N** | 0.03 | -0.12 | -0.01 | -0.03 | 0.11 | -0.02 | 0.02 | -0.03 | 0.02 | -0.18 | -0.17 | 0.03 | 0.06 | 0.21 | 1 |
