# Supplementary material for: Nitrogen Use Efficiency in Sorghum: Exploring Native Variability for Traits Under Variable N-Regimes
Source: Front Plant Sci. 2021 Apr 21;12:643192. doi: 10.3389/fpls.2021.643192 (PMC8097177; doi:10.3389/fpls.2021.643192)
Supplement: Supplementary Table 5 — Correlation coefficient analysis of 60 genotypes at N0 (A), N50 (B), and N100 (C) dosages for 2017–2018 at ICRISAT, Patancheru. [file Table_5.DOCX]

**Supplementary table 5:** Correlation coefficient analysis of 60 genotypes at N0 (5A), N50 (5B) and N100 (5C) dosage for 2017-2018 at ICRISAT, Patancheru.

5A

**5B**

**5C**
